# Learning curve analysis of applying Seprafilm hyaluronic acid/carboxymethylcellulose membrane during laparoscopic hysterectomy

**DOI:** 10.1038/s41598-020-73692-y

**Published:** 2020-10-06

**Authors:** Yi-Ting Huang, Yu-Ying Su, Kai-Yun Wu, Hui-Yu Huang, Yu-Shan Lin, Cindy Hsuan Weng, Lan-Yan Yang, Yu-Bin Pan, Chin-Jung Wang

**Affiliations:** 1 Department of Obstetrics and Gynecology, Chang Gung Memorial Hospital At Linkou, Taoyuan, Taiwan; 2 Chang Gung University College of Medicine, Taoyuan, Taiwan; 3 Department of Obstetrics and Gynecology, Chang Gung Memorial Hospital, Taipei, Taiwan; 4 Biostatistics Unit, Clinical Trial Center, Chang Gung Memorial Hospital, Kweishan, Taoyuan, Taiwan; 5 Gynecologic Cancer Research Center, Chang Gung Memorial Hospital, Kweishan, Taoyuan, Taiwan

**Keywords:** Urogenital reproductive disorders, Outcomes research

## Abstract

This study was designed to evaluate the learning curve of applying Seprafilm (modified hyaluronic acid and carboxymethylcellulose; Genzyme, Cambridge, MA, USA) during laparoscopic hysterectomy or subtotal hysterectomy with or without adnexectomy. In this retrospective cohort study, 35 patients who underwent laparoscopic hysterectomy or subtotal hysterectomy with or without adnexectomy were enrolled. The Seprafilm was cut into 4 pieces, rolled up with a trimmed plastic sleeve and delivered through an incision wound made for the 5-mm ancillary trocar. The membrane was unrolled and placed on the rough surface after hysterectomy or subtotal hysterectomy with or without adnexectomy. The time from the insertion of the first piece of membrane into the abdominal cavity to the complete removal of the trimmed plastic sleeve was recorded. The median time for Seprafilm placement was 3 min. The learning curve was analyzed using the power-law method and suggested that 10 cases were required to achieve proficiency in the procedure. The presence of adnexectomy was significantly associated with the time required for Seprafilm placement (*P* < 0.001). Although Seprafilm placement is more complicated compared to the liquid and gel forms of anti-adhesion barriers, surgical proficiency seemed to be attained after 10 cases for an experienced surgeon.

## Introduction

Minimizing scarring and cosmetic damage during surgery is crucial for both external and internal body parts. Postoperative adhesion after abdominal or pelvic surgery is a type of interior scaring which leads to bowel obstruction, chronic abdominal pain, and female infertility and increases complication risk during future surgeries. Adhesiolysis involves advanced surgical technique and increased surgical time; intra- and post-operative complication risk, such as inadvertently incurred bowel defects; the incidence of sepsis; and postoperative hospital stay^1^.

Different types of adhesion barriers have been used since the nineteenth century. The first commercially available adhesion barrier was Cargile Membrane, a sterile membrane made from ox’s peritoneum^2^. Interceed (Gynecare, Ethicon, a Johnson & Johnson Company, Somerville, NJ, USA), Seprafilm (Genzyme, Cambridge, MA, USA), Hyalobarrier Gel (Fidia Advanced Biopolymers, Abano Terme, Italy), Adept (Icodextrin 4% solution; Baxter Healthcare, Deerfield, IL, USA), and BarriGel (HANBIO, New Taipei City, Taiwan) are the currently available adhesion barriers in our hospital, used after gynecologic and obstetric surgery. Except for Interceed, which is an oxidized regenerated cellulose membrane, these barriers come in either gel or liquid form and are easy to handle during laparoscopic surgery^3^. However, Seprafilm is composed of two anionic polysaccharides, modified hyaluronic acid and carboxymethylcellulose, which makes it brittle and causes it to adhere to moist surfaces such as surrounding tissues and surgical instruments ultimately leading to a high placement failure rate. Several different techniques have been published on the insertion of Seprafilm^4–9^; however, none has investigated the associated learning curve of applying this barrier after laparoscopic surgery.

The learning curve is a graphic representation that shows the decrease in the time consumed or improvement in surgical skills (on the vertical axis) when performing a similar task repeatedly (horizontal axis). During medical education and training, the learning curve helps to recommend the number of cases a surgeon must practice on in simulation before they can treat real patients. The result of the learning curve varies among surgeons and depends on task complexity, surgical procedure, and institutional factors. This study aimed to evaluate the learning curve of applying Seprafilm during laparoscopic hysterectomy or subtotal hysterectomy with or without adnexectomy by a single surgeon using the power-law method.

## Materials and methods

This retrospective study recruited 35 women (age range 41–60 years, median 47 years) with symptomatic uterine fibroids, adenomyosis, or endometrial hyperplasia scheduled to undergo laparoscopic hysterectomy or subtotal hysterectomy with or without adnexectomy performed by a single surgeon (CJW) with more than 15 years of experience in performing advanced laparoscopic gynecologic procedures at the Chang Gung Memorial Hospital in Linkou. Bilateral salpingectomy was performed simultaneously since 2016 after the procedure was advised as primary prevention for ovarian cancer^10^. Unilateral salpingo-oophorectomy, bilateral salpingo-oophorectomy, or cystectomy were performed if a benign adnexal mass was diagnosed pre-operatively. Written informed consent was obtained from all the patients after a thorough explanation of the use of Seprafilm.

Preoperative clinical and demographic characteristics, including the patient’s age, body mass index (BMI), and parity from each group, were collected. The overall operating time, estimated blood loss, presence of adnexectomy, weight of the specimen, time spent placing the Seprafilm, and duration of postoperative hospital stay were recorded. Seprafilm placement time was defined as the time from the insertion of the first piece of Seprafilm into the abdominal cavity until the complete removal of the last trimmed plastic sleeve. The study was approved by the Institutional Review Board of Chang Gung Medical Foundation (institutional review board no: 201901139B0); relevant guidelines and regulations were followed accordingly.

### Surgical techniques

**Figure 1:**
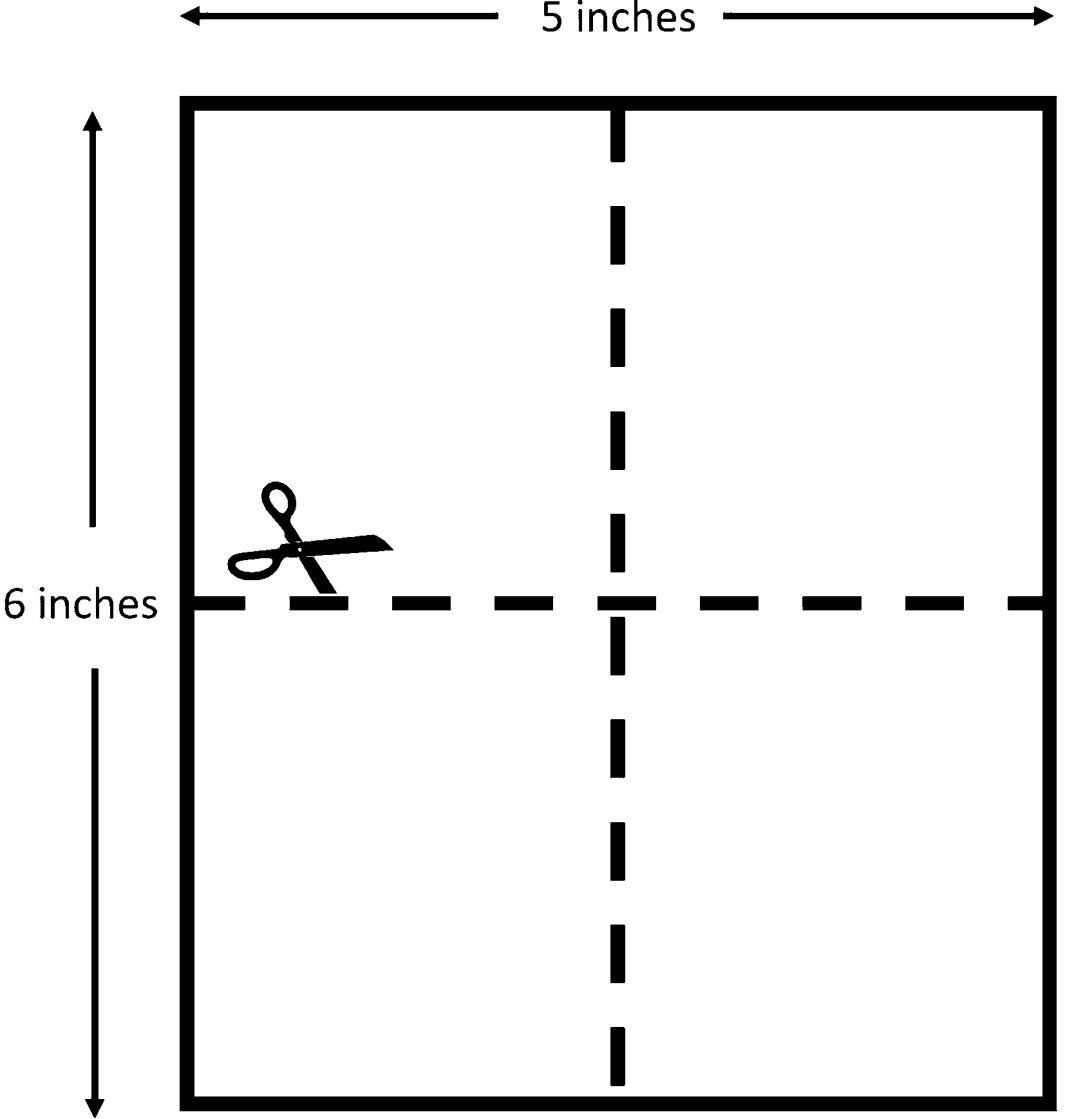
One piece of Seprafilm (5 in. × 6 in.) was cut into 4 equally sized pieces.

**Figure 2:**
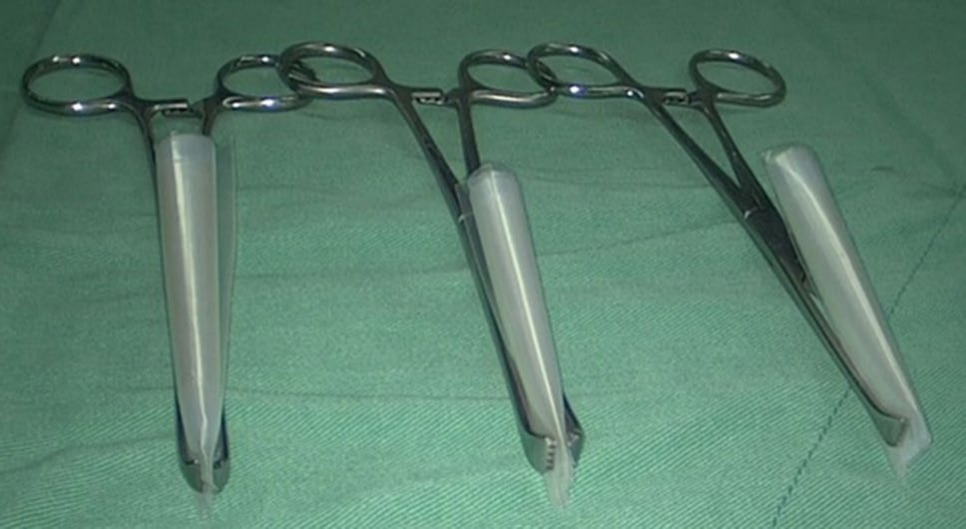
A single piece of Seprafilm is placed in the middle of a trimmed plastic sleeve and rolled up into a tube-shaped roll. One end of the roll was gripped with Allis tissue forceps.

Laparoscopic hysterectomy or subtotal hysterectomy were performed following the 4-trocar technique: the 5-mm principal trocar introduced through the umbilicus, one 5-mm cannula in the right lower quadrant lateral to the inferior epigastric arteries, one 5-mm cannula in the left lower quadrant, and one 5 mm was made in the left abdomen at the paramedian line at the level of the umbilicus. After the procedure, the Seprafilm was unwrapped and left on the surgical trolley for 2 min to absorb moisture from the surrounding air. Once softened, the Seprafilm and a Tyvek, a white paper that protects both sides of the Seprafilm before spreading (Fig. 1), were cut into 4 equally sized pieces with scissors. A sterile plastic sleeve (GMTH, New Taipei City, Taiwan) that was used to wrap the camera head and fiber optic light cable, was measured and cut slightly larger than the size of the single piece of Seprafilm. The Tyvek was removed and placed on one single piece of Seprafilm in the middle of the trimmed plastic sleeve and rolled up into a tube-shaped roll. The time consumed by the scrubbing nurse to prepare the Seprafilm from 1 piece to 4 rolls on the surgical trolley was about 5 min. Once prepared, one end of the roll was gripped with an Allis tissue forceps (Fig. 2) and inserted into the abdominal cavity through the incision wound after the 5-mm ancillary trocar (left-upper) was removed. The transversalis fascia layer was dilated with an Allis tissue forceps in cases where insertion of the tube-shaped roll was difficult.

The surgical assistant used forceps to grasp the edge of the trimmed plastic sleeve and the surgeon unrolled it, also using forceps, exposing the Seprafilm. A further pair of forceps was used to hold the Seprafilm in place and adhere it directly to the postoperative traumatized surface in the pelvic cavity. The trimmed plastic sleeve was removed, and the next piece of Seprafilm was inserted using the same method until all of the Seprafilm covered the rough surface areas completely. Finally, the trimmed plastic sleeve was removed from the peritoneum cavity. A small amount of saline solution was irrigated through the Seprafilm to enhance the attachment if necessary.

### Statistical analysis

The learning curves of applying Seprafilm were measured as the time consumed during the placement of Seprafilm over the time course of the study. The power-law equation was used to analyze the learning curves in this study. The simplest and most used mathematical form of power law in this type of study is T = a P^b^^11^, where T is the Seprafilm placement time, parameter a is the curve’s starting point, P is a practice measure (patient’s sequential number) and parameter b is the skill improvement rate. As the slope of the power-law asymptotically approaches 0, the point at which it crosses − 1.0 was defined as the threshold for proficiency.

Discrete probability distributions or continuous probability distributions data were expressed as median (range). Univariate and multivariable linear models were developed based on the variables (age, parity, body mass index, and adnexectomy) to assess the single and joint effects of the variables on each outcome of interest, respectively. Two-sided *P* values less than 0.05 were considered statistically significant. Statistical analyses were performed using the PASW Statistics for Windows, version 18.0 (SPSS, Inc., Chicago, IL, USA).

## Results

Among the 35 women, 31 hysterectomies and four subtotal hysterectomies with or without adnexectomy were performed. The median age at the time of surgery was 47 years, median BMI was 25 kg/m^2^, and the median of parity was 2 (Table 1). The median weight of the uterus with or without adnexa surgery was 380 g. Out of the 35 cases, 15 (42.9%) underwent the adnexectomy procedure. The median operating time was 100 min, none of the cases required blood transfusion during or after the surgery with a median blood loss of 150 mL during the surgeries. The median of time consumed during Seprafilm placement was 3 min and none of the cases failed. The median of postoperative hospital stay was 2 days. No complications, such as postoperative pelvic abscess formation, ureter, bladder, or bowel injury, occurred.

**Table 1:** Patient characteristics and surgical outcomes (N = 35).

*Patient characteristics*	
Age (years)	47 (41.60)
Parity, median (range)	2 (0.3)
Body mass index (kg/m^2^)	25 (20.35)
Specimen weight (g)	380 (95.1130)
Adnexectomy	
Yes	15 (42.9%)
No	20 (57.1%)
*Surgical outcomes*	
Operating time (min)	100 (40.240)
Blood loss (mL)	150 (30.950)
Seprafilm placement time (min)	3 (2.4)
Failure for Seprafilm placement	0 (0%)
Hospital stay (days)	2 (2.3)

Data are presented as median (range) or n (%).

The details of the univariate analyses of the association between demography/presence of adnexectomy and the time consumed in Seprafilm placement are shown in Table 2. The presence of adnexectomy was observed to be significantly associated with the time consumed during Seprafilm placement (*P* < 0.001). The same result was seen in the multivariate regression analysis (*P* < 0.001) (Table 3).

**Table 2:** Univariable analysis of characteristics of patients for Seprafilm placement time.

Parameter	Univariate			
	Beta	Standard error	95% CI	*P* value
Age (years)	− 0.027	0.018	− 0.063 to 0.008	0.129
Parity	− 0.054	0.071	− 0.198 to 0.091	0.456
Body mass index (kg/m^2^)	− 0.008	0.026	− 0.060 to 0.044	0.756
Adnexectomy	− 0.7	0.114	− 0.933 to − 0.467	< 0.001

*CI* confidence interval.

**Table 3:** Multivariable analysis of characteristics of patients for Seprafilm placement time.

Parameter	Multivariate			
	Beta	Standard error	95% CI	*P* value
Age (years)	− 0.016	0.013	− 0.042 to 0.009	0.201
Parity	− 0.049	0.049	− 0.149 to 0.052	0.333
Body mass index (kg/m^2^)	0.012	0.018	− 0.025 to 0.049	0.509
Adnexectomy	− 0.702	0.116	− 0.94 to − 0.465	< 0.001

*CI* confidence interval.

**Figure 3:**
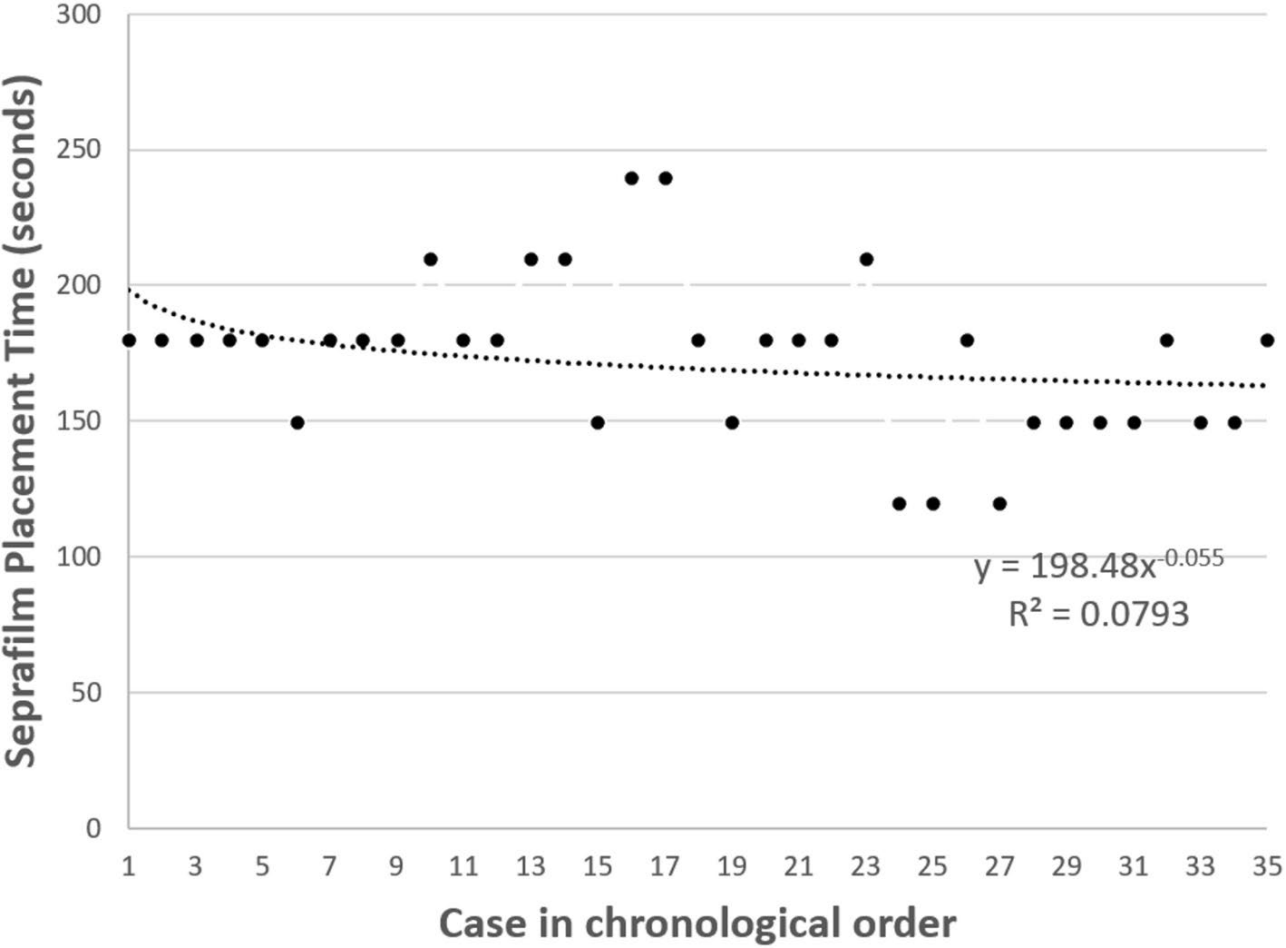
The trend line was calculated using the power-law equation: (Seprafilm placement time) = 198.48 ×  (patient’s sequential number) ^ (− 0.055) with an R^2^ = 0.0793. The proficient point occurs at Case 10.

Furthermore, the trend lines of the time consumed in Seprafilm placement in seconds were plotted against the patients’ sequential in chronological order to demonstrate if a learning curve could be observed. The trend line fitted the function of power-law equation: (Seprafilm placement time) = 198.48 × (patient’s sequential number) ^ (− 0.055) with an R^2^ = 0.0793. An apparent decreasing trend was seen for the values of the initial cases and asymptotically approaches 0; the point at which it crossed − 1.0 was defined as the threshold for proficiency, and this occurred at the value for the 10th case (Fig. 3).

## Discussion

Seprafilm is brittle and easily adheres to moist surfaces such as surrounding tissues and surgical instruments. Therefore, before cutting it into pieces, Seprafilm was left on a surgical trolley for 2 min to absorb moisture from the air, which softens it and makes it more flexible. Different methods have been previously used for moistening Seprafilm^7,9^. Moreover, before unrolling the trimmed plastic sleeve and exposing the Seprafilm, all instruments required to be wiped clean first and forceps had to be dry when in contact with the membrane. In our study, instead of passing the Seprafilm through the 11 mm or 12 mm trocar during the insertion into the peritoneal cavity^8,9^, we removed the 5 mm ancillary trocar and inserted the membrane roll through the incision wound directly. This method avoids the Seprafilm becoming stuck inside the trocar sheath and allows the use of a smaller trocar resulting in a smaller incision wound during multi-port laparoscopic surgery.

There are several methods for Seprafilm placement, including (1) dissolving it in warm normal saline, which gives Seprafilm solution^12^; (2) wrapping it around the Endpeanut cylindrically, which creates a Seprafilm flag^5^; (3) using an introducer (Reduction Sleeve; KARL STORZ GmbH & Co.KG, Tuttlingen, Germany)^8^ or adhesion prevention sheet (APS) introducer (Hakko, Tokyo, Japan)^13^; (4) backloading into the exteriorized trocar with a protective paper sheath^4^;(5) rolling up Seprafilm with the backing from the package and inserting it through the 11 mm trocar sleeve^9^. All of these techniques require either additional instruments or larger trocar sleeves compared to the one used in our study. The mean total time needed for Seprafilm placement reported by Kusuki et al.^7^ was 601 ± 248 s but only 16/67 patients (23.9%) underwent a laparoscopic hysterectomy in that study. The mean application time for Seprafilm placement in single-port and multiport laparoscopic surgery reported by Hong et al.^9^ was 4.0 ± 1.47 min but the result includes the addition of application times for laparoscopic myomectomy, total/subtotal hysterectomy, sacrocolpopexy, cystectomy, and cancer surgery. Our study is the first to record the time consumed in Seprafilm placement during laparoscopic hysterectomy or subtotal hysterectomy with or without adnexectomy, and the median time was 3 min per surgery.

Certainly, the more proficient the surgeon on the laparoscopic technique, the more rapid the reduction in a learning curve of new laparoscopic procedures^14^. Demographic/perioperative variables might also play roles in affecting surgeons’ performance on a new technique. The influence on the operative time by specimen weight^15^ and the size of the adnexal mass^16^ has been reported. Therefore, univariate and multivariable linear models were developed based on the variables (age, parity, body mass index, and adnexectomy) to assess the single and joint effects of the variables on Seprafilm placement time. The specimen weight (uterus) was not taken into account in this study because the placement of Seprafilm was performed after laparoscopic hysterectomy or subtotal hysterectomy. The time consumed in Seprafilm placement was associated with the adnexectomy procedure as shown in Tables 2 and 3. The requirement for adnexectomy decreased the time needed for Seprafilm placement. This may be due to the absence of organs that cause interference, as Seprafilm placement becomes much easier on a flat surface. This can be verified in the report of Takeuchi et al.^13^, in which higher difficulty and failure rate were observed on uneven surfaces.

In this study, one piece of Seprafilm was divided equally into 4 pieces instead of using one full piece or dividing it in half, because a larger piece leads to an increased degree of difficulty in placement, especially inside a confined space. Seprafilm easily adheres to adjacent organs, especially the bowel, during placement and manipulation. Moreover, Seprafilm can be divided into more than 4 pieces. A smaller Seprafilm size will help to overcome the problem of undesired adherence to surrounding tissues during manipulation; however, this will increase the total placement time compared to that required for 4 pieces. After several attempts, we found that dividing Seprafilm into 4 pieces was the most appropriate approach.

A sterile plastic sleeve (GMTH, New Taipei City, Taiwan), prevents Seprafilm pieces from sticking together after being rolled up into a tube-shaped roll. Moreover, it also protects the membrane while being inserted into the peritoneal cavity through a 5-mm incision wound and prevents adherence to undesired sites. This method has decreased the failure rate of Seprafilm placement, for instance, no failures occurred in our study compared to that of Takeuchi et al.^13^ that reported 87/702 (12.4%) cases of damaged Seprafilm.

During the study period, we found that extensive surgery was needed when the patient had adenomyosis accompanied with deep infiltrating endometriosis (DIE) that invaded surrounding tissues. DIE is a highly invasive form of endometriosis that would cause fibrosis in adjacent tissues and cause them to adhere to each other, making surgical procedures complicated^17^. The rough surfaces caused by extensive surgery during hysterectomy and excision of endometriotic foci may increase the risk of postoperative adhesion formation. Thus, the application of an adhesion-prevention barrier such as Seprafilm would be appropriate and beneficial.

The strengths of this study included the analytic approach and the lack of confounders between different surgeons or institutions which may influence the result. This study had several limitations. First, we did not perform second-look laparoscopy to ensure the integrity of Seprafilm and the effect of Seprafilm as an anti-adhesion barrier. Second, all surgeries in this study were performed and recorded by the same surgeon (CJW), who was an experienced laparoscopist. That means the results may not apply to inexperienced surgeons (resident doctors) and the number of cases required to achieve proficiency in Seprafilm placement during laparoscopic hysterectomy/subtotal hysterectomy may differ between surgeons.

In conclusion, although Seprafilm placement is more difficult than the placement of liquid and gel form anti-adhesion barriers, surgical proficiency seemed to be attained after 10 cases for an experienced surgeon. The presence of adnexectomy was significantly associated with the time consumed in Seprafilm placement as an anti-adhesion barrier after laparoscopic hysterectomy or subtotal hysterectomy.
